# 4-Nitro­phenyl naphthalene-1-sulfonate

**DOI:** 10.1107/S1600536808027396

**Published:** 2008-08-30

**Authors:** Jasmine P. Vennila, Helen P. Kavitha, D. John Thiruvadigal, B. R. Venkatraman, V. Manivannan

**Affiliations:** a Department of Physics, Panimalar Institute of Technology, Chennai 600 095, India; b Department of Chemistry, SRM University, Ramapuram, Chennai 600 089, India; c Department of Physics, SRM University, Kattankulathur Campus, Chennai, India; d Department of Chemistry, Periyar E.V.R. College, Tiruchirappalli 620 023, India; e Department of Physics, Presidency College, Chennai 600 005, India

## Abstract

In the crystal structure of the title compound, C_16_H_11_NO_5_S, the plane of the naphthalene ring system forms a dihedral angle of 63.39 (8)° with the benzene ring. The nitro group makes a dihedral angle of 10.73 (16)° with the benzene ring. Weak intra- and inter­molecular C—H⋯O inter­actions are observed.

## Related literature

For related literature, see: Manivannan *et al.* (2005 ▶); Vennila *et al.* (2008 ▶); Yachi *et al.* (1989 ▶).
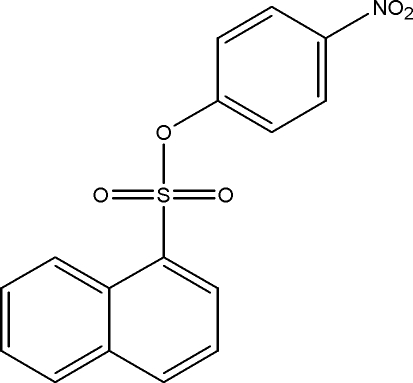


## Experimental

### 

#### Crystal data

C_16_H_11_NO_5_S
*M* *_r_* = 329.32
Monoclinic, 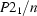

*a* = 13.4407 (8) Å
*b* = 6.2990 (3) Å
*c* = 18.2556 (12) Å
β = 106.296 (2)°
*V* = 1483.48 (15) Å^3^
*Z* = 4
Mo *K*α radiation
μ = 0.24 mm^−1^
*T* = 295 (2) K
0.28 × 0.20 × 0.16 mm

#### Data collection

Bruker Kappa APEXII diffractometer
Absorption correction: multi-scan (**SADABS**; Sheldrick, 1996 ▶) *T*_min_ = 0.935, *T*_max_ = 0.962
14281 measured reflections
2801 independent reflections
1935 reflections with *I* > 2σ(*I*)
*R*_int_ = 0.041

#### Refinement

*R*[*F* ^2^ > 2σ(*F* ^2^)] = 0.046
*wR*(*F* ^2^) = 0.160
*S* = 1.08
2801 reflections
208 parameters
H-atom parameters constrained
Δρ_max_ = 0.25 e Å^−3^
Δρ_min_ = −0.20 e Å^−3^

### 

Data collection: *APEX2* and *SAINT* (Bruker, 2004 ▶); cell refinement: *APEX2*; data reduction: *APEX2*; program(s) used to solve structure: *SHELXS97* (Sheldrick, 2008 ▶); program(s) used to refine structure: *SHELXL97* (Sheldrick, 2008 ▶); molecular graphics: *PLATON* (Spek, 2003 ▶); software used to prepare material for publication: *SHELXL97*.

## Supplementary Material

Crystal structure: contains datablocks I, global. DOI: 10.1107/S1600536808027396/is2328sup1.cif

Structure factors: contains datablocks I. DOI: 10.1107/S1600536808027396/is2328Isup2.hkl

Additional supplementary materials:  crystallographic information; 3D view; checkCIF report

## Figures and Tables

**Table 1:** Hydrogen-bond geometry (Å, °)

*D*—H⋯*A*	*D*—H	H⋯*A*	*D*⋯*A*	*D*—H⋯*A*
C8—H8⋯O2	0.93	2.43	2.829 (5)	106
C16—H16⋯O3	0.93	2.46	3.058 (4)	122
C5—H5⋯O2^i^	0.93	2.48	3.179 (3)	132
C15—H15⋯O4^ii^	0.93	2.47	3.340 (5)	155

Symmetry codes: (i) 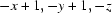; (ii) 
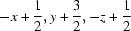
.

**Fig. 1.**
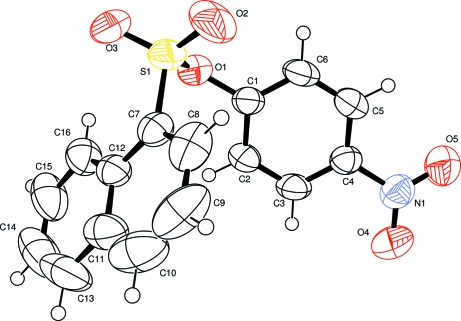
The molecular structure of (I), with atom labels and 50% probability displacement ellipsoids for non-H atoms.

**Fig. 2.**
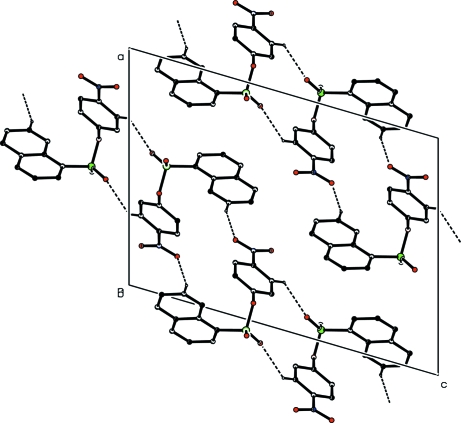
The packing of (I), viewed down the b axis. Hydrogen bonds are shown as dashed lines. H atoms not involved in hydrogen bonding have been omitted.

## supplementary crystallographic information

### Comment

The merging of lipids can be monitored using a derivative of *para*-toluene sulfonate (Yachi *et al.*, 1989). The geometric parameters of the title molecule, (I) (Fig. 1), agree well with the reported structures (Manivannan *et al.* 2005; Vennila *et al.* 2008)

The plane of the benzene ring forms a dihedral angle of 63.39 (8)° with the naphthalene ring system. The torsion angles O2—S1—C7—C8 and O3—S1—C7—C12 [0.1 (3) ° and 45.8 (2)°, respectively] indicate the *syn* conformation of sulfonyl moiety. The molecular structure is stabilized by weak intramolecular C—H···O interactions and the crystal packing is stabilized by weak intermolecular C—H···O interactions (Fig. 2).

### Experimental

1-Naphthalene sulfonyl chloride (2.5 mmol) dissolved in acetone (4 ml) was added dropwise to 4-nitrophenol (2.5 mmol) in aqueous NaOH (4 ml, 5%) with constant shaking. The precipitated compound (1.8 mmol, yield 72%) was recrystallized from ethanol to get diffraction quality pale yellow crystals.

### Refinement

H atoms were positioned geometrically and refined using riding model, with C—H = 0.93 Å, and with *U*_iso_(H) = 1.2*U*_eq_(C).

### Crystal data

**Table:**

C_16_H_11_NO_5_S	*F*_000_ = 680
*M**_r_* = 329.32	*D*_x_ = 1.474 Mg m^−^^3^
Monoclinic, *P*2_1_/*n*	Mo *K*α radiation λ = 0.71073 Å
Hall symbol: -P 2yn	Cell parameters from 4818 reflections
*a* = 13.4407 (8) Å	θ = 2.2–25.4º
*b* = 6.2990 (3) Å	µ = 0.24 mm^−^^1^
*c* = 18.2556 (12) Å	*T* = 295 (2) K
β = 106.296 (2)º	Block, yellow
*V* = 1483.48 (15) Å^3^	0.28 × 0.20 × 0.16 mm
*Z* = 4	

### Data collection

**Table:**

Bruker Kappa APEX2 diffractometer	2801 independent reflections
Radiation source: fine-focus sealed tube	1935 reflections with *I* > 2σ(*I*)
Monochromator: graphite	*R*_int_ = 0.041
*T* = 295(2) K	θ_max_ = 25.7º
ω and φ scans	θ_min_ = 1.7º
Absorption correction: multi-scan(*SADABS*; Sheldrick, 1996)	*h* = −16→16
*T*_min_ = 0.935, *T*_max_ = 0.962	*k* = −7→5
14281 measured reflections	*l* = −22→22

### Refinement

**Table:**

Refinement on *F*^2^	Secondary atom site location: difference Fourier map
Least-squares matrix: full	Hydrogen site location: inferred from neighbouring sites
*R*[*F*^2^ > 2σ(*F*^2^)] = 0.046	H-atom parameters constrained
*wR*(*F*^2^) = 0.160	*w* = 1/[σ^2^(*F*_o_^2^) + (0.0801*P*)^2^ + 0.4092*P*] where *P* = (*F*_o_^2^ + 2*F*_c_^2^)/3
*S* = 1.08	(Δ/σ)_max_ < 0.001
2801 reflections	Δρ_max_ = 0.25 e Å^−^^3^
208 parameters	Δρ_min_ = −0.20 e Å^−^^3^
Primary atom site location: structure-invariant direct methods	Extinction correction: none

### Fractional atomic coordinates and isotropic or equivalent isotropic displacement parameters (Å^2^)

**Table:**

	*x*	*y*	*z*	*U*_iso_*/*U*_eq_	
S1	0.54713 (6)	0.94373 (13)	0.12153 (4)	0.0704 (3)	
C1	0.37278 (18)	0.7401 (4)	0.09748 (13)	0.0528 (6)	
C2	0.3303 (2)	0.7033 (4)	0.15655 (14)	0.0598 (6)	
H2	0.3391	0.8008	0.1961	0.072*	
C3	0.2749 (2)	0.5218 (4)	0.15633 (15)	0.0613 (7)	
H3	0.2454	0.4937	0.1957	0.074*	
C4	0.26342 (18)	0.3817 (4)	0.09726 (14)	0.0537 (6)	
C5	0.3065 (2)	0.4177 (4)	0.03838 (15)	0.0654 (7)	
H5	0.2979	0.3198	−0.0010	0.079*	
C6	0.3621 (2)	0.5999 (4)	0.03865 (15)	0.0673 (7)	
H6	0.3921	0.6277	−0.0005	0.081*	
C7	0.58144 (18)	0.8465 (4)	0.21516 (15)	0.0576 (6)	
C8	0.6346 (2)	0.6587 (5)	0.2287 (2)	0.0839 (9)	
H8	0.6500	0.5851	0.1891	0.101*	
C9	0.6658 (3)	0.5785 (7)	0.3043 (4)	0.1117 (16)	
H9	0.7032	0.4525	0.3147	0.134*	
C10	0.6415 (3)	0.6838 (9)	0.3611 (3)	0.1157 (15)	
H10	0.6627	0.6289	0.4103	0.139*	
C11	0.5863 (2)	0.8702 (6)	0.34821 (18)	0.0846 (10)	
C12	0.55403 (18)	0.9590 (4)	0.27417 (14)	0.0577 (6)	
C13	0.5581 (4)	0.9799 (11)	0.4083 (2)	0.1245 (18)	
H13	0.5799	0.9270	0.4579	0.149*	
C14	0.5005 (4)	1.1579 (11)	0.3943 (3)	0.137 (2)	
H14	0.4815	1.2248	0.4337	0.165*	
C15	0.4701 (3)	1.2404 (7)	0.3225 (3)	0.1029 (12)	
H15	0.4312	1.3647	0.3139	0.124*	
C16	0.4949 (2)	1.1465 (5)	0.26351 (18)	0.0725 (8)	
H16	0.4725	1.2067	0.2151	0.087*	
O1	0.42304 (14)	0.9358 (3)	0.09765 (10)	0.0644 (5)	
O2	0.58335 (18)	0.7992 (4)	0.07547 (14)	0.1056 (8)	
O3	0.56871 (18)	1.1638 (4)	0.11968 (12)	0.0901 (7)	
O4	0.1795 (2)	0.1450 (4)	0.15547 (16)	0.1094 (9)	
O5	0.1800 (2)	0.0787 (4)	0.04028 (14)	0.0949 (7)	
N1	0.20241 (18)	0.1888 (4)	0.09722 (15)	0.0705 (6)	

### Atomic displacement parameters (Å^2^)

**Table:**

	*U*^11^	*U*^22^	*U*^33^	*U*^12^	*U*^13^	*U*^23^
S1	0.0768 (5)	0.0848 (6)	0.0604 (5)	−0.0147 (4)	0.0373 (4)	−0.0034 (3)
C1	0.0608 (14)	0.0552 (14)	0.0448 (12)	0.0025 (11)	0.0187 (10)	0.0049 (11)
C2	0.0746 (16)	0.0619 (16)	0.0507 (14)	0.0009 (12)	0.0304 (12)	−0.0052 (12)
C3	0.0741 (16)	0.0690 (17)	0.0507 (15)	0.0013 (13)	0.0337 (12)	0.0051 (12)
C4	0.0581 (14)	0.0527 (14)	0.0511 (14)	0.0055 (11)	0.0168 (11)	0.0068 (11)
C5	0.0781 (17)	0.0737 (18)	0.0491 (14)	−0.0039 (14)	0.0254 (13)	−0.0083 (12)
C6	0.0809 (18)	0.085 (2)	0.0436 (14)	−0.0105 (15)	0.0306 (13)	−0.0030 (13)
C7	0.0517 (13)	0.0586 (15)	0.0676 (16)	−0.0013 (12)	0.0252 (12)	0.0026 (12)
C8	0.0627 (16)	0.0708 (19)	0.124 (3)	0.0023 (15)	0.0362 (18)	−0.0020 (19)
C9	0.065 (2)	0.092 (3)	0.167 (5)	0.0121 (17)	0.014 (2)	0.057 (3)
C10	0.080 (2)	0.154 (4)	0.098 (3)	−0.011 (3)	0.001 (2)	0.056 (3)
C11	0.0654 (18)	0.120 (3)	0.0612 (19)	−0.0228 (19)	0.0052 (14)	0.0139 (18)
C12	0.0504 (13)	0.0711 (17)	0.0525 (15)	−0.0091 (12)	0.0158 (11)	0.0008 (12)
C13	0.101 (3)	0.224 (6)	0.0479 (19)	−0.057 (3)	0.0186 (19)	−0.009 (3)
C14	0.119 (4)	0.214 (6)	0.096 (4)	−0.058 (4)	0.058 (3)	−0.076 (4)
C15	0.095 (2)	0.116 (3)	0.112 (3)	−0.019 (2)	0.052 (2)	−0.053 (2)
C16	0.0740 (18)	0.0747 (19)	0.0751 (19)	−0.0021 (15)	0.0312 (15)	−0.0112 (15)
O1	0.0746 (12)	0.0641 (12)	0.0561 (11)	−0.0065 (9)	0.0206 (9)	0.0071 (8)
O2	0.1001 (16)	0.145 (2)	0.0946 (16)	−0.0191 (15)	0.0642 (14)	−0.0393 (15)
O3	0.1088 (17)	0.0895 (15)	0.0763 (14)	−0.0348 (12)	0.0328 (12)	0.0139 (11)
O4	0.151 (2)	0.0943 (17)	0.1024 (19)	−0.0314 (15)	0.0678 (18)	0.0109 (14)
O5	0.1127 (19)	0.0819 (16)	0.0837 (17)	−0.0223 (12)	0.0167 (13)	−0.0090 (12)
N1	0.0763 (15)	0.0610 (15)	0.0735 (17)	0.0015 (11)	0.0196 (13)	0.0074 (13)

### Geometric parameters (Å, °)

**Table:**

S1—O2	1.415 (2)	C8—C9	1.418 (6)
S1—O3	1.419 (2)	C8—H8	0.9300
S1—O1	1.602 (2)	C9—C10	1.346 (6)
S1—C7	1.751 (3)	C9—H9	0.9300
C1—C6	1.367 (4)	C10—C11	1.374 (6)
C1—C2	1.374 (3)	C10—H10	0.9300
C1—O1	1.405 (3)	C11—C12	1.414 (4)
C2—C3	1.364 (4)	C11—C13	1.435 (6)
C2—H2	0.9300	C12—C16	1.406 (4)
C3—C4	1.368 (4)	C13—C14	1.345 (7)
C3—H3	0.9300	C13—H13	0.9300
C4—C5	1.375 (4)	C14—C15	1.361 (7)
C4—N1	1.466 (3)	C14—H14	0.9300
C5—C6	1.369 (4)	C15—C16	1.350 (4)
C5—H5	0.9300	C15—H15	0.9300
C6—H6	0.9300	C16—H16	0.9300
C7—C8	1.368 (4)	O4—N1	1.218 (3)
C7—C12	1.422 (4)	O5—N1	1.215 (3)
			
O2—S1—O3	120.53 (15)	C9—C8—H8	120.6
O2—S1—O1	108.88 (12)	C10—C9—C8	120.3 (4)
O3—S1—O1	103.19 (12)	C10—C9—H9	119.9
O2—S1—C7	108.34 (15)	C8—C9—H9	119.9
O3—S1—C7	111.42 (13)	C9—C10—C11	121.7 (4)
O1—S1—C7	102.92 (10)	C9—C10—H10	119.1
C6—C1—C2	122.2 (2)	C11—C10—H10	119.1
C6—C1—O1	120.9 (2)	C10—C11—C12	120.5 (4)
C2—C1—O1	116.9 (2)	C10—C11—C13	121.8 (4)
C3—C2—C1	119.1 (2)	C12—C11—C13	117.7 (4)
C3—C2—H2	120.5	C16—C12—C11	118.3 (3)
C1—C2—H2	120.5	C16—C12—C7	124.8 (2)
C2—C3—C4	118.9 (2)	C11—C12—C7	116.9 (3)
C2—C3—H3	120.5	C14—C13—C11	121.1 (4)
C4—C3—H3	120.5	C14—C13—H13	119.5
C3—C4—C5	122.1 (2)	C11—C13—H13	119.5
C3—C4—N1	118.3 (2)	C13—C14—C15	120.3 (4)
C5—C4—N1	119.6 (2)	C13—C14—H14	119.9
C6—C5—C4	119.0 (2)	C15—C14—H14	119.9
C6—C5—H5	120.5	C16—C15—C14	121.7 (4)
C4—C5—H5	120.5	C16—C15—H15	119.1
C1—C6—C5	118.8 (2)	C14—C15—H15	119.1
C1—C6—H6	120.6	C15—C16—C12	120.9 (3)
C5—C6—H6	120.6	C15—C16—H16	119.5
C8—C7—C12	121.8 (3)	C12—C16—H16	119.5
C8—C7—S1	117.4 (2)	C1—O1—S1	119.41 (15)
C12—C7—S1	120.8 (2)	O5—N1—O4	123.7 (3)
C7—C8—C9	118.8 (4)	O5—N1—C4	118.6 (2)
C7—C8—H8	120.6	O4—N1—C4	117.7 (3)
			
C6—C1—C2—C3	−0.5 (4)	C13—C11—C12—C16	−1.2 (4)
O1—C1—C2—C3	175.8 (2)	C10—C11—C12—C7	0.1 (4)
C1—C2—C3—C4	0.1 (4)	C13—C11—C12—C7	−179.1 (3)
C2—C3—C4—C5	0.4 (4)	C8—C7—C12—C16	−176.5 (3)
C2—C3—C4—N1	−179.1 (2)	S1—C7—C12—C16	3.0 (3)
C3—C4—C5—C6	−0.3 (4)	C8—C7—C12—C11	1.3 (4)
N1—C4—C5—C6	179.1 (2)	S1—C7—C12—C11	−179.13 (19)
C2—C1—C6—C5	0.5 (4)	C10—C11—C13—C14	−177.4 (4)
O1—C1—C6—C5	−175.6 (2)	C12—C11—C13—C14	1.8 (6)
C4—C5—C6—C1	−0.1 (4)	C11—C13—C14—C15	−1.7 (7)
O2—S1—C7—C8	0.1 (3)	C13—C14—C15—C16	0.9 (7)
O3—S1—C7—C8	−134.7 (2)	C14—C15—C16—C12	−0.3 (5)
O1—S1—C7—C8	115.3 (2)	C11—C12—C16—C15	0.5 (4)
O2—S1—C7—C12	−179.42 (19)	C7—C12—C16—C15	178.3 (3)
O3—S1—C7—C12	45.8 (2)	C6—C1—O1—S1	−78.7 (3)
O1—S1—C7—C12	−64.2 (2)	C2—C1—O1—S1	104.9 (2)
C12—C7—C8—C9	−2.0 (4)	O2—S1—O1—C1	54.4 (2)
S1—C7—C8—C9	178.4 (2)	O3—S1—O1—C1	−176.40 (18)
C7—C8—C9—C10	1.3 (5)	C7—S1—O1—C1	−60.4 (2)
C8—C9—C10—C11	0.1 (6)	C3—C4—N1—O5	170.0 (3)
C9—C10—C11—C12	−0.8 (5)	C5—C4—N1—O5	−9.4 (4)
C9—C10—C11—C13	178.4 (4)	C3—C4—N1—O4	−12.0 (4)
C10—C11—C12—C16	178.0 (3)	C5—C4—N1—O4	168.5 (3)

### Hydrogen-bond geometry (Å, °)

**Table:**

*D*—H···*A*	*D*—H	H···*A*	*D*···*A*	*D*—H···*A*
C8—H8···O2	0.93	2.43	2.829 (5)	106
C16—H16···O3	0.93	2.46	3.058 (4)	122
C5—H5···O2^i^	0.93	2.48	3.179 (3)	132
C15—H15···O4^ii^	0.93	2.47	3.340 (5)	155

Symmetry codes: (i) −*x*+1, −*y*+1, −*z*; (ii) −*x*+1/2, *y*+3/2, −*z*+1/2.
